# Comparison of anterior segment parameter measurements between Sirius Plus and Pentacam AXL

**DOI:** 10.3389/fmed.2026.1819141

**Published:** 2026-04-21

**Authors:** Jiliang Ning, Lijun Zhang

**Affiliations:** 1 Department of Ophthalmology, The Third People’s Hospital of Dalian, Dalian, China; 2 Department of Ophthalmology, Dalian Third People’s Hospital Affiliated to Dalian University of Technology, Dalian, China

**Keywords:** agreement, biometry, Pentacam, Scheimpflug imaging, Sirius

## Abstract

**Introduction:**

This cross-sectional study compared the differences, consistencies, and correlations of the measurements of anterior segment biological parameters between Sirius Plus and Pentacam AXL in a healthy population with myopia.

**Methods:**

Eighty patients with myopia (80 eyes) scheduled for refractive surgery at the Ophthalmology Department of the Third People’s Hospital of Dalian from December 2024 to June 2025 were included. One eye was randomly selected for each participant, and measurements were taken using Sirius Plus and Pentacam AXL. Fourteen parameters related to the anterior segment were extracted.

**Results:**

The differences in the measurements for anterior flat keratometry, anterior vertical/horizontal astigmatism, and anterior oblique astigmatism at 45° between the two devices were not significant, whereas those for the other parameters were significant. The intraclass correlation coefficient values for anterior flat keratometry, anterior steep keratometry, and white-to-white were 0.965, 0.963, and 0.932, respectively. The goodness-of-fit analysis showed that the *R*^2^ values for the anterior flat keratometry, anterior steep keratometry, central corneal thickness, white-to-white, corneal volume, and anterior chamber depth were all >0.85, which demonstrated a strong correlation.

**Discussion:**

Anterior corneal keratometry and white-to-white had good correlation and consistency, indicating they are suitable mutual reference points for clinical practice.

## 1 Introduction

Ophthalmic biometry data are of critical importance for corneal refractive surgery and phakic intraocular lens implantation. Precise measurements of corneal curvature, corneal thickness, and corneal diameter are essential for surgical accuracy. Post-refractive corneal ectasia is a sight-threatening complication, and accurate preoperative screening for keratoconus or subclinical keratoconus helps to improve surgical safety (1). Various measuring devices have emerged, including slit scanning, Scheimpflug topography, Placido discs, IOLMaster and swept-source optical coherence tomography. Owing to differences in the measurement principles of these devices, the results obtained can vary (2–5). The Pentacam (Oculus, Wetzlar, Germany) and Sirius (CSO, Florence, Italy) are two of the most common devices available on the market for obtaining anterior segment measurements. The Pentacam (Oculus, Wetzlar, Germany) and Sirius (CSO, Florence, Italy) are two of the most common devices available on the market for obtaining anterior segment measurements. Compared with IOLMaster or anterior segment OCT devices, Pentacam and Sirius offer refractive analysis modules, including for keratoconus screening and corneal aberration analysis. The Pentacam utilizes a rotating Scheimpflug camera to capture high-resolution tomographic images of the anterior segment, whereas the Sirius employs a combination of the Placido disc and Scheimpflug camera rotation principles to obtain parameters of the anterior segment (6, 7). Sirius Plus and Pentacam AXL are the latest models of these devices. These upgraded data acquisition modules can obtain more anterior segment data points and achieve faster acquisition than their predecessors.

Several studies have investigated the consistency of measurements for anterior segment parameters, such as corneal curvature, corneal thickness, corneal volume, and anterior chamber depth, between the Sirius and Pentacam HR; however, previous findings have been contradictory (8–11). However, to date, no research has compared anterior segment parameters obtained between the latest models, the Sirius Plus and Pentacam AXL.

In this study, we measured the anterior segment biological parameters in healthy individuals with myopia using Sirius Plus and Pentacam AXL and subsequently compared the differences, consistencies, and correlations between the two instruments to provide references for clinical applications.

## 2 Methods

### 2.1 Research participants

In this retrospective cross-sectional study, 80 patients (80 eyes) who were scheduled for refractive surgery and underwent preoperative examinations at the Ophthalmology Department of the Dalian Third People’s Hospital from December 2024 to June 2025 were included; one eye from each patient was randomly selected for the analyses. Data collection for research purposes began on September 1, 2025. The average age of the participants was 22.93 ± 5.53 years; 41 males and 39 females. The best corrected visual acuity of the participants was at least 20/20, and they had discontinued wearing soft contact lenses for more than 2 weeks prior to the study. Patients with active eye diseases; history of eye surgery or trauma, corneal scarring, glaucoma, or keratoconus; autoimmune diseases, or other systemic diseases affecting the eyes; or women who were pregnant or breastfeeding were excluded.

This study adhered to the Declaration of Helsinki and was approved by the Ethics Committee of the Dalian Third People’s Hospital (Approval No.: 2025-183-001). In accordance with the requirements of the ethics committee, a waiver regarding obtaining signed informed consent from the patients was granted.

### 2.2 Equipment for examination

The Sirius Plus anterior segment analysis system integrates a Placido disc projection with 22 rings and Scheimpflug camera rotation imaging technology. The Scheimpflug camera performs 360-degree rotational photography of the anterior segment using visible blue light at a wavelength of 475 nm, capturing 25/50/100 corneal cross-sectional and anterior chamber images within 3 s. This system can measure 42,032–151,232 points on the anterior cornea and 36,400–145,600 points on the posterior corneal surface, thereby provide comprehensive anterior segment parameters including corneal curvature, corneal thickness, corneal height, and anterior chamber depth, within a 12 mm diameter range.

The Pentacam AXL anterior segment analysis system employs Scheimpflug rotational scanning technology using visible light (blue light) at 475 nm for rotational photography and can collect 25, 50, or 100 anterior segment profile images within 2 s. It can measure up to 138,000 data points and generate precise three-dimensional models of the anterior chamber and cornea.

### 2.3 Examination method

All examinations were performed by the same examiner. To avoid the influence of external light and circadian rhythms, all checks were performed in a dark room without windows between 10:00 and 14:00. Measurements were taken in random order using the Sirius Plus and Pentacam AXL devices, with a 10-min interval between the two devices. Before measurement, the participants were instructed to blink several times to ensure stability of the tear film. The results with the highest collection quality and without significant eyelid obstruction were selected and included in the analyses to ensure data stability. Fifteen anterior segment parameters, including corneal curvature, corneal geometric features, and anterior chamber parameters, were included in the analyses (Table 1).

**Table 1:** Description of the anterior segment parameters.

Abbreviation	Parameter definition
Anterior flat K	Corneal curvature on the flat meridian of the anterior corneal surface within a 3 mm diameter
Anterior steep K	Corneal curvature on the steep meridian of the anterior corneal surface within a 3 mm diameter
Anterior J0	Vertical/horizontal astigmatic component of the anterior corneal surface within a 3 mm diameter
Anterior J45	Oblique astigmatic component at 45° of the anterior corneal surface within a 3 mm diameter
Posterior flat K	Corneal curvature on the flat meridian of the posterior corneal surface within a 3 mm diameter
Posterior steep K	Corneal curvature on the steep meridian of the posterior corneal surface within a 3 mm diameter
Posterior J0	Vertical/horizontal astigmatic component of the posterior corneal surface within a 3 mm diameter
Posterior J45	Oblique astigmatic component at 45° of the posterior corneal surface within a 3 mm diameter
CCT	Central corneal thickness
WTW	Horizontal corneal white-to-white diameter
CV	Corneal volume within a 10 mm diameter
ACD	Anterior chamber depth
ACA	Horizontal mean iridocorneal angle
ACV	Anterior chamber volume within a 12 mm diameter region

K, keratometry.

### 2.4 Statistical analyses

Statistical analyses of the data were performed using the MedCalc 22.001 software (MedCalc Software Ltd., Ostend, Belgium). Quantitative data are presented as mean ± standard deviation and range. The Shapiro–Wilk test and histograms were used to verify whether the data met the criteria for normal distribution or approximate normality. Paired *t*-tests were used to compare differences in the anterior segment parameters measured using different devices. The reliability of the two devices was assessed using the intraclass correlation coefficient (ICC) and a 95% confidence interval (CI). Bland–Altman analysis was performed to evaluate the consistency of measurements between the Sirius Plus and Pentacam AXL devices. The 95% limits of agreement were calculated as the mean difference ± 1.96 times the standard deviation of the differences and used as the consistency evaluation index. A linear regression analysis was conducted to assess correlations among the parameters across devices, and the coefficient of determination (*R*^2^) was computed. Corneal curvature was used as the primary observation index for the sample size calculation. Based on preliminary experimental results, the maximum allowable difference between the methods was set at 0.45, with a significance level of 0.05 and a statistical power of 0.80, yielding a minimum required sample size of 79 eyes. Significance was set at *p* < 0.05.

## 3 Results

This study included 80 participants (80 eyes) (41 males and 39 females) with an average age of 22.93 ± 5.53 years. One eye from each patient was randomly selected for analyses. The results of the differential analysis for the anterior segment parameters measured using the Sirius Plus and Pentacam AXL are presented in Table 2. For the average anterior flat keratometry, the measurements from Sirius Plus and Pentacam AXL were 42.14 ± 1.37 diopters (D) and 42.14 ± 1.37 D, respectively, with no significant difference between the two (*p* = 0.544). For the average anterior steep keratometry (anterior steep K), the Sirius Plus measurement was 43.54 ± 1.35 D, whereas Pentacam AXL’s measurement was 43.63 ± 1.44 D, which showed a significant difference (*p* = 0.047); however, this difference lacks clinical significance. For the vector decomposition of corneal anterior surface astigmatism, the parameters anterior vertical/horizontal astigmatism (anterior J0) and anterior oblique astigmatism at 45°(anterior J45) were measured; Sirius Plus reported averages of 0.64 ± 0.34 D and −0.02 ± 0.27 D, and Pentacam AXL reported averages of 0.64 ± 0.34 D and −0.02 ± 0.27 D, respectively, without a significant difference (all *p* > 0.05). Measurements for the remaining anterior segment parameters obtained with the two devices differed significantly (*p* < 0.05).

**Table 2:** Differences in anterior segment parameter measurements between Sirius Plus and Pentacam AXL.

Parameter	Sirius Plus		Pentacam AXL		*t*	*p*
	Mean ± SD	Range	Mean ± SD	Range		
Anterior flat K, D	42.14 ± 1.37	38.79, 45.06	42.17 ± 1.33	39, 45.6	−0.609	0.544
Anterior steep K, D	43.54 ± 1.35	40.43, 46.98	43.63 ± 1.44	40.5, 47.3	−2.016	0.047
Anterior J0, D	0.64 ± 0.34	−0.1, 1.77	0.69 ± 0.4	−0.12, 2.41	−1.941	0.056
Anterior J45, D	−0.02 ± 0.27	−0.78, 1.39	−0.02 ± 0.23	−0.52, 0.97	−0.153	0.879
Posterior flat K, D	5.89 ± 0.21	5.31, 6.4	6.06 ± 0.22	5.5, 6.6	−15.083	<0.001
Posterior steep K, D	6.28 ± 0.23	5.75, 6.8	6.49 ± 0.24	6, 7.1	−19.9	<0.002
Posterior J0, D	−0.18 ± 0.06	−0.33, −0.03	−0.22 ± 0.07	−0.46, −0.05	5.97	<0.003
Posterior J45, D	−0.04 ± 0.05	−0.14, 0.07	0 ± 0.06	−0.2, 0.14	−6.273	<0.004
CCT, μm	544.08 ± 29.57	458, 607	552.63 ± 28.71	470, 613	−10.616	<0.006
WTW, mm	11.7 ± 0.39	10.76, 12.58	11.76 ± 0.39	10.8, 12.7	−4.446	<0.007
CV, mm^3^	57.72 ± 3.09	49.7, 64.4	62.04 ± 3.05	53.1, 68.8	−40.647	<0.008
ACD, mm	3.28 ± 0.21	2.84, 3.79	3.2 ± 0.22	2.69, 3.73	14.047	<0.009
ACA, °	49.43 ± 3.14	41.8, 56	42.36 ± 5.76	27.8, 56.9	12.917	<0.010
ACV, mm^3^	200.81 ± 23.88	139.02, 257.66	190.56 ± 26.2	133, 255	4.93	<0.011

ACA, anterior chamber angle; ACD, anterior chamber depth; ACV, anterior chamber volume; CCT, central corneal thickness; CV, corneal volume; D, diopters; K, keratometry; SD, standard deviation; WTW, white-to-white.

**Figure 1:**
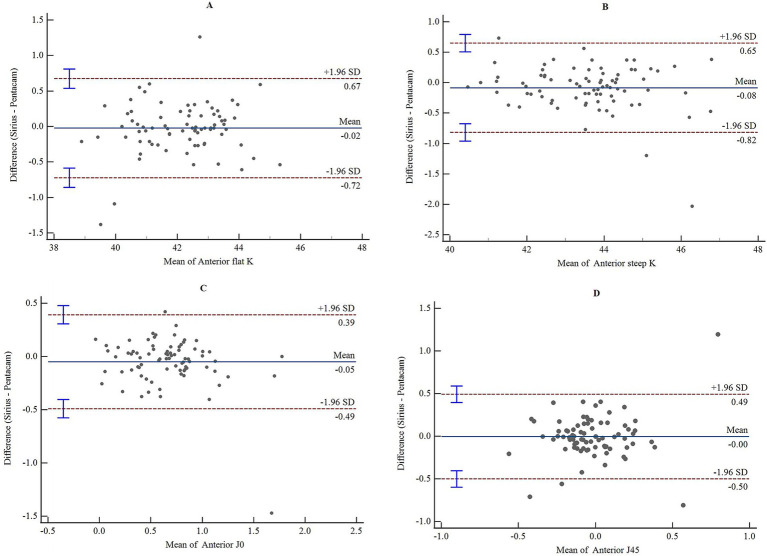
Bland–Altman diagram illustrating the pairwise comparison of anterior flat K, anterior steep K, anterior J0, and anterior J45 between Sirius Plus and Pentacam AXL **(A)** Anterior flat K; **(B)** Anterior steep K; **(C)** Anterior J0; **(D)** Anterior J45. Anterior J0, vertical/horizontal astigmatic component of the anterior corneal surface within a 3 mm diameter; J45, oblique astigmatic component at 45° of the anterior corneal surface within a 3 mm diameter; K, keratometry.

**Figure 2:**
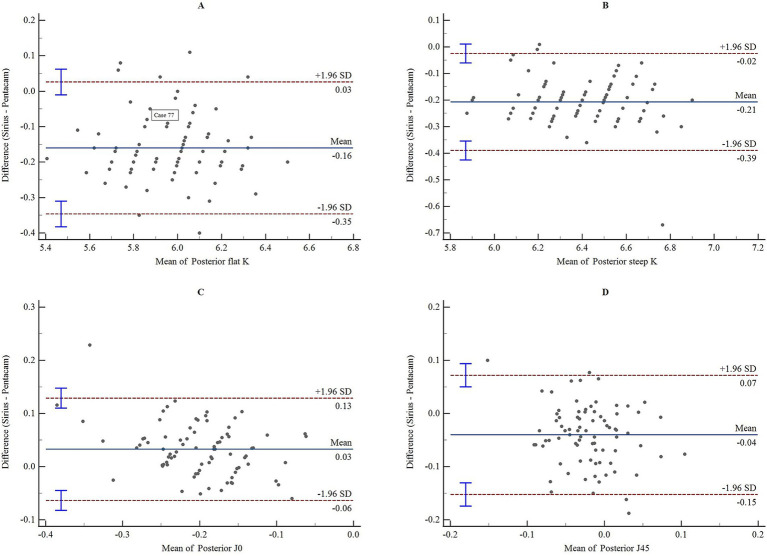
Bland–Altman diagram illustrating the pairwise comparison of posterior flat K, posterior steep K, posterior J0, and posterior J45 between Sirius Plus and Pentacam AXL **(A)** Posterior flat K; **(B)** Posterior steep K; **(C)** Posterior J0; **(D)** Posterior J45. Posterior J0, vertical/horizontal astigmatic component of the posterior corneal surface within a 3 mm diameter; J45, oblique astigmatic component at 45° of the posterior corneal surface within a 3 mm diameter; K, keratometry.

**Figure 3:**
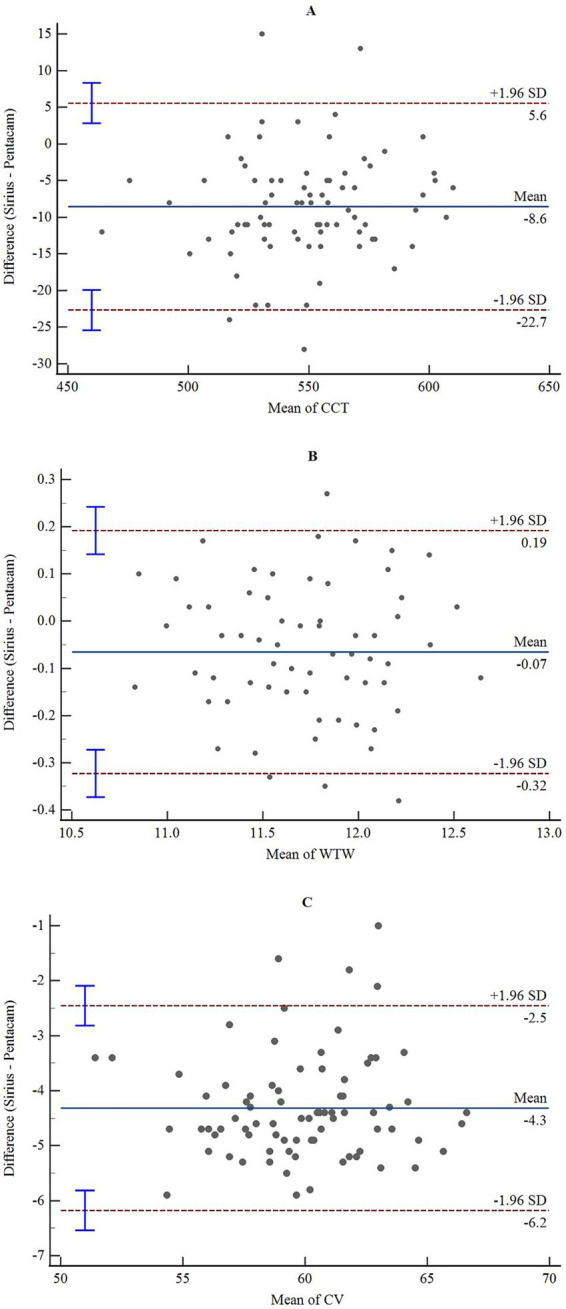
Bland–Altman diagram illustrating the pairwise comparison of CCT, WTW, and CV between Sirius Plus and Pentacam AXL **(A)** CCT; **(B)** WTW; **(C)** CV. CCT, central corneal thickness; WTW, white-to-white; CV, corneal volume.

**Figure 4:**
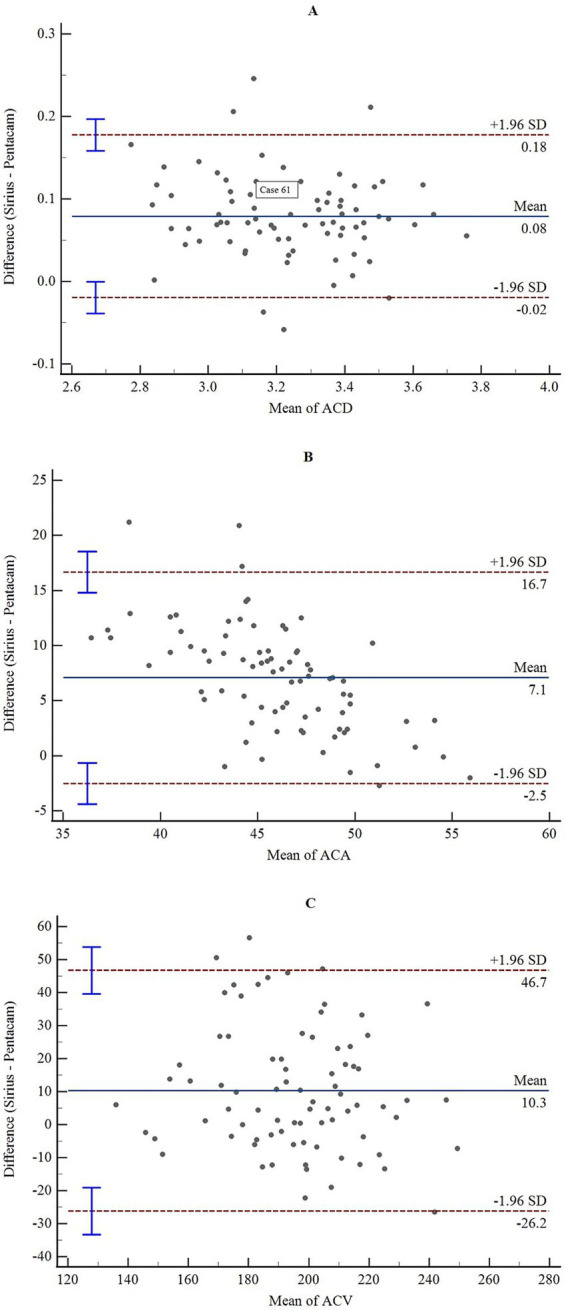
Bland–Altman diagram illustrating the pairwise comparison of ACD, ACA, and ACV between Sirius Plus and Pentacam AXL **(A)** ACD; **(B)** ACA; **(C)** ACV. ACD, anterior chamber depth; ACA, anterior chamber angle; ACV, nterior chamber volume.

The consistency and correlation analyses of the anterior segment parameters measured by Sirius Plus and Pentacam AXL are presented in Table 3. Measurements of the anterior flat keratometry (anterior flat K) and anterior steep K using the two devices showed excellent consistency, with ICC values of 0.965 (0.947–0.978) and 0.963 (0.942–0.976), respectively. The interdevice consistency for the anterior J0 showed moderate-to-good agreement, with an ICC of 0.812 (0.720–0.876). For white-to-white (WTW), consistency across devices was rated as good to excellent, with an ICC of 0.932 (0.861–0.962). For the remaining parameters, the lower limits of the 95% CI for the inter-device ICC were all <0.5, indicating poor reliability. Bland–Altman plots are presented in Figures 1–4 and show the agreement between Sirius Plus and Pentacam AXL for the anterior segment parameters, with 95% limits of agreement.

**Table 3:** Consistency and correlation analysis of anterior segment parameter measurements between Sirius Plus and Pentacam AXL.

Parameter	ICC	95% CI	*R* ^2^	*p*
Anterior flat K, D	0.965	0.947–0.978	0.94	<0.001
Anterior steep K, D	0.963	0.942–0.976	0.94	<0.001
Anterior J0, D	0.812	0.720–0.876	0.69	<0.001
Anterior J45, D	0.481	0.292–0.633	0.23	<0.001
Posterior flat K, D	0.705	−0.007 to 0.906	0.81	<0.001
Posterior steep K, D	0.661	−0.067 to 0.896	0.85	<0.001
Posterior J0, D	0.656	0.332–0.812	0.56	<0.001
Posterior J45, D	0.289	0.026–0.504	0.14	<0.001
CCT, μm	0.930	0.422–0.978	0.94	<0.001
WTW, mm	0.932	0.861–0.962	0.88	<0.001
CV, mm^3^	0.479	−0.021 to 0.821	0.90	<0.001
ACD, mm	0.913	0.115–0.976	0.94	<0.001
ACA, °	0.206	−0.091 to 0.487	0.28	<0.001
ACV, mm^3^	0.671	0.427–0.806	0.53	<0.001

ACA, anterior chamber angle; ACD, anterior chamber depth; ACV, anterior chamber volume; CCT, central corneal thickness; CI, confidence interval; CV, corneal volume; D, diopters; ICC, intraclass correlation coefficient; K, keratometry; WTW, white to white.

**Figure 5:**
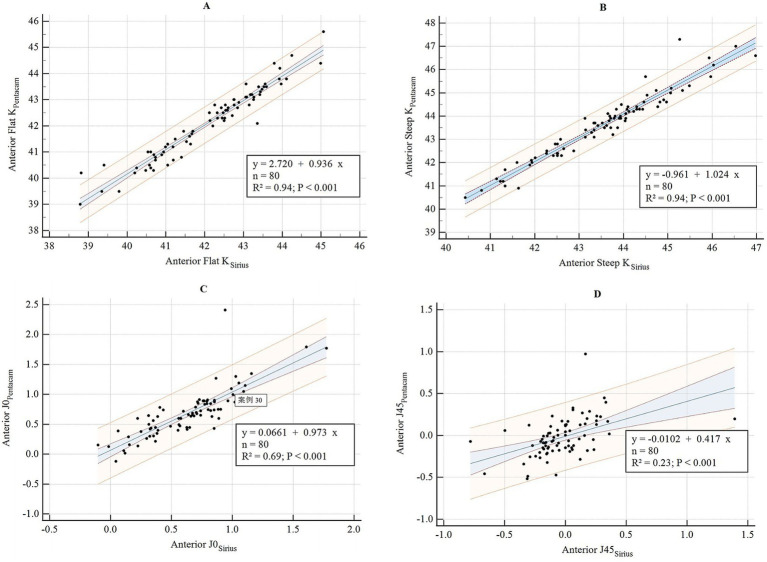
Correlation analysis of anterior flat K, anterior steep K, anterior J0, and anterior J45 between Sirius Plus and Pentacam AXL **(A)** Anterior flat K; **(B)** Anterior steep K; **(C)** Anterior J0; **(D)** Anterior J45. Anterior J0, vertical/horizontal astigmatic component of the anterior corneal surface within a 3 mm diameter; J45, oblique astigmatic component at 45° of the anterior corneal surface within a 3 mm diameter; K, keratometry.

**Figure 6:**
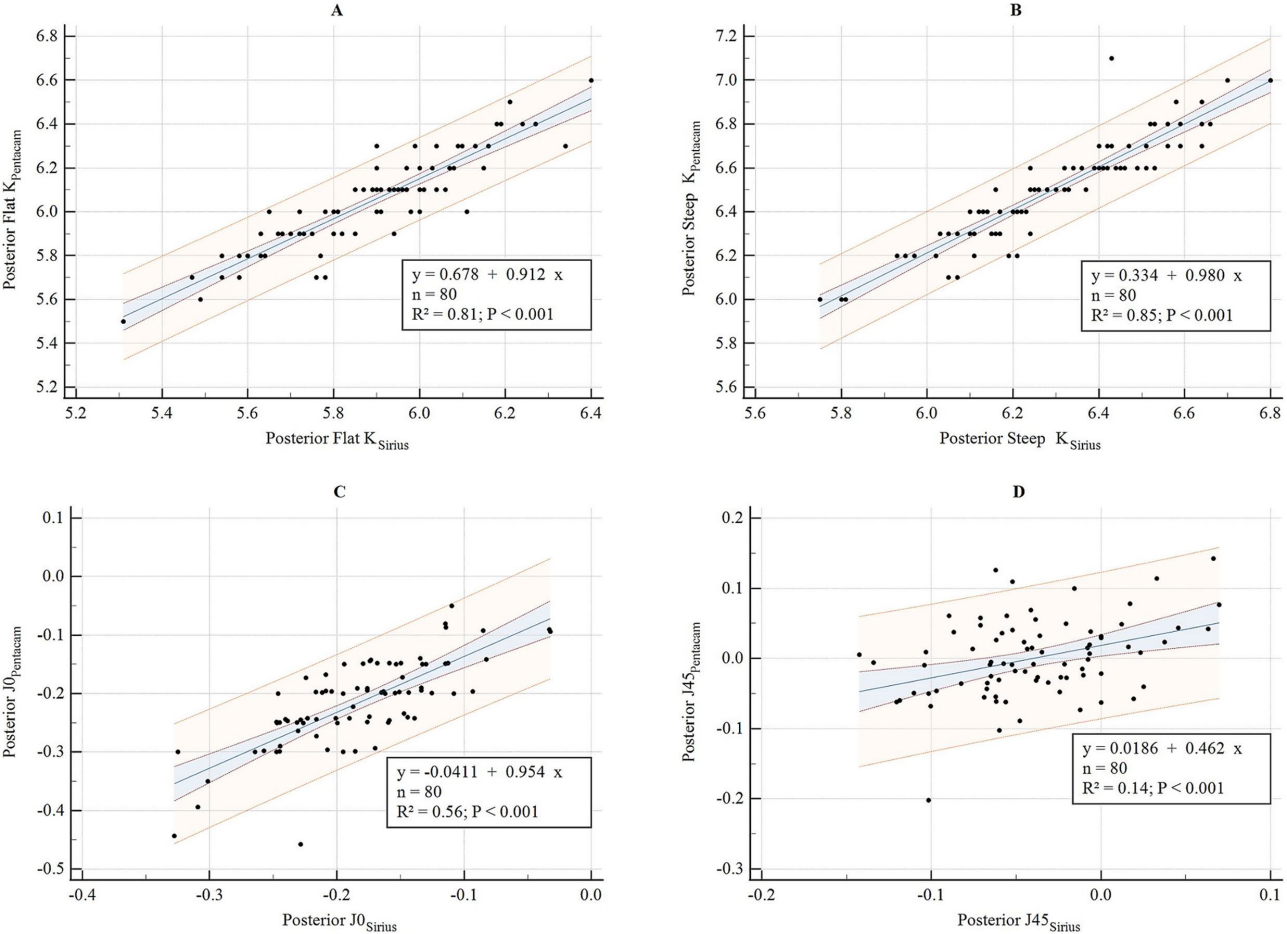
Correlation analysis of posterior flat K, posterior steep K, posterior J0, and posterior J45 between Sirius Plus and Pentacam AXL **(A)** Posterior flat K; **(B)** Posterior steep K; **(C)** Posterior J0; **(D)** Posterior J45. Posterior J0, vertical/horizontal astigmatic component of the posterior corneal surface within a 3 mm diameter; J45, oblique astigmatic component at 45° of the posterior corneal surface within a 3 mm diameter; K, keratometry.

**Figure 7:**
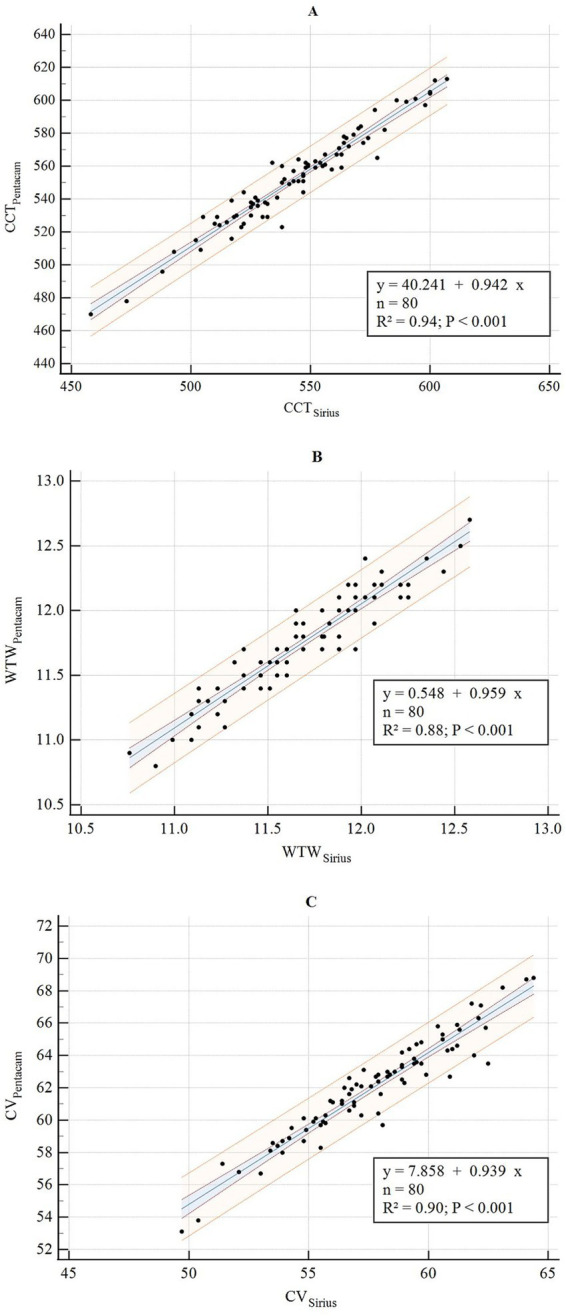
Correlation analysis of CCT, WTW, and CV between Sirius Plus and Pentacam AXL **(A)** CCT; **(B)** WTW; **(C)** CV. CCT, central corneal thickness; WTW, white-to-white; CV, corneal volume.

**Figure 8:**
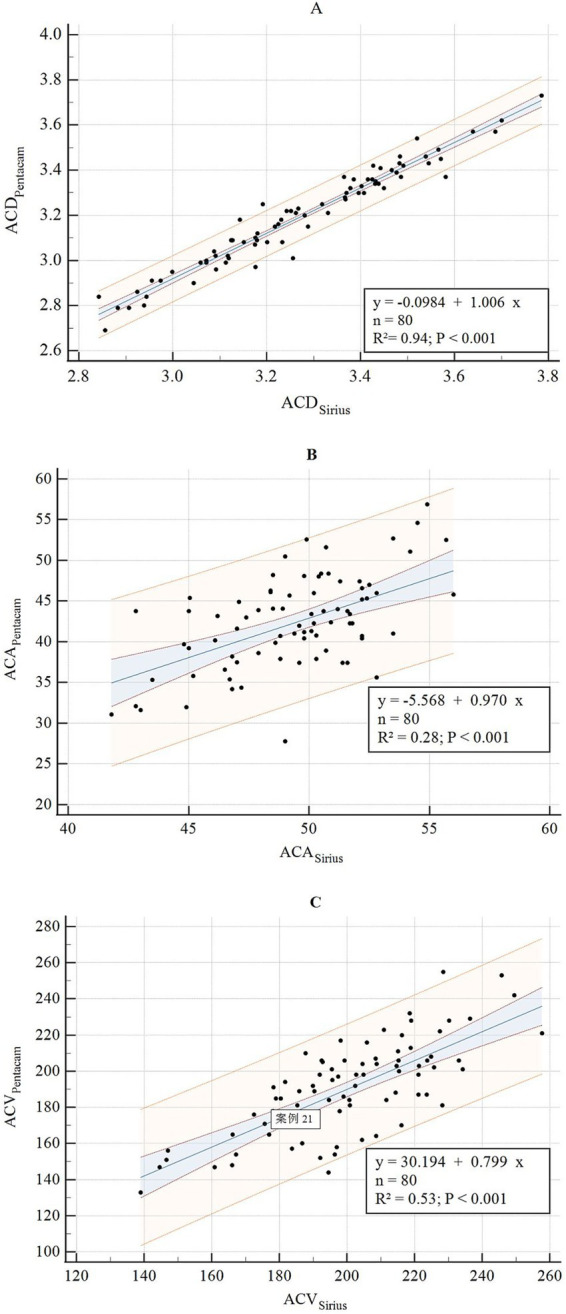
Correlation analysis of ACD, ACA, and ACV between Sirius Plus and Pentacam AXL **(A)** ACD; **(B)** ACA; **(C)** ACV. ACD, anterior chamber depth; ACA, anterior chamber angle; ACV, anterior chamber volume.

The results of the linear regression for the anterior segment parameters measured using both devices are listed in Table 3 and Figures 5–8. The parameters anterior flat K, anterior steep K, central corneal thickness, WTW, corneal volume, and anterior chamber depth (ACD), with goodness-of-fit values greater than 0.85, had *R*^2^ values of 0.94, 0.94, 0.94, 0.88, 0.90, and 0.94, respectively (all *p* < 0.001).

## 4 Discussion

The Pentacam and Sirius possess robust measurement capabilities, which enable analysis of multiple parameters ranging from the anterior corneal surface to the lens. Thus, they are the most commonly used anterior segment analysis devices in clinical practice (12). Medical institutions are typically equipped with only one of these two devices. When patients visit different institutions, ophthalmologists must verify the compatibility of the two devices to enable effective comparison of relevant examination parameters. Previous studies have primarily focused on analyzing ≥1 parameter, such as corneal curvature, anterior chamber depth, and corneal thickness. In this study, we systematically compared the measurement characteristics of 14 anterior segment parameters in a healthy population using two new anterior segment analysis devices, Sirius Plus and Pentacam AXL (8, 13, 14).

The differing measurement principles of the two devices may partially explain the observed discrepancies in anterior segment parameters. Pentacam AXL relies solely on a rotating Scheimpflug camera, which captures cross-sectional images of the anterior segment and reconstructs three-dimensional models based on tomographic data. In contrast, Sirius Plus combines Placido disk-based corneal topography with Scheimpflug tomography. This disk provides high-resolution curvature data of the anterior corneal surface, whereas the Scheimpflug camera contributes to posterior corneal surface and internal structure measurements. Differences in acquisition speed and point density may affect measurement stability, particularly in the presence of microsaccades or tear film instability.

The measurement of corneal curvature and astigmatism, particularly of the anterior corneal surface, plays a crucial role in the diagnosis and treatment of ocular diseases. These include diagnosis of keratoconus, fitting of corneal contact lenses, postoperative predictions for refractive surgery, and planning of intraocular lenses for cataract surgery (15–17). This study results demonstrated no significant differences between the Sirius Plus and Pentacam AXL in measuring the curvature and astigmatism of the anterior corneal surface, specifically in anterior flat K, anterior J0, and anterior J45. Although there was a significant difference in anterior steep K, this difference was not clinically significant.

Both devices exhibited excellent consistency and correlation when the curvature of the anterior corneal surface was measured. Han et al. utilized Pentacam, Sirius, and IOLMaster 700 to measure 252 patients with myopia and found good agreement in the consistency of flat K, steep K, J0, and J45 (13). Savini et al. conducted corneal topography measurements on 25 patients and found that the measurements of the anterior corneal surface curvature by Sirius and Pentacam showed minimal differences, with a narrow limit of agreement ranging from −0.59 to +0.59 D at 95% (18). We observed significant differences in posterior corneal surface curvature and astigmatism parameters, with poor consistency. Therefore, only the anterior surface curvature is considered interchangeable in clinical practice.

Accurate measurement of the corneal thickness is crucial for determining the feasibility of surgical methods in laser corneal refractive surgery, selecting the type of laser procedure, and predicting safe ablation depth (19). Errors in assessing corneal thickness may lead to anomalies in the corneal ablation depth and residual stromal thickness, thereby increasing the risk of postoperative iatrogenic corneal ectasia (20). The average CCT measured in this study using Sirius Plus was 544.08 ± 29.57 μm, which is consistent with previous findings (8, 9, 21). Bayhan et al. used four devices to measure the CCT in 50 patients. The average CCT measured by the Sirius device was 525.92 ± 34.10 μm, which is consistent with our results (22). Soulantzou et al. measured the average CCT of 216 volunteers using Pentacam HR, reporting a value of 547.31 ± 35.28 μm, which is relatively consistent with the measurement of 552.63 ± 28.71 μm obtained in this study (23). Han et al. found that the average CCT values measured by the Pentacam and Sirius showed no significant differences and exhibited high correlation (8).

Our results indicated that although the average CCT measurements from the two anterior segment analysis devices were well correlated, there was a significant difference, with Sirius Plus measuring an average CCT 8.05 μ lower than Pentacam AXL, and the 95% limits of agreement were −22.7 to 5.6 μm. Therefore, the devices are not recommended to be used interchangeably in clinical practice.

WTW is the horizontal visible iris diameter, which plays a crucial role in the fitting of contact lenses, planning of refractive surgery, and implantation of intraocular lenses. Currently, WTW measurement methods are divided into manual caliper measurements and automated measurements (IOLMaster or Orbscan topography systems) (24). The measurement methods of Sirius and Pentacam utilize an iris camera photographic method that automatically identifies the distance between the two iris edges on the horizontal meridian in the grayscale image (25, 26). Gharieb et al. found that for healthy participants, the WTW measurements obtained with Sirius were higher compared with those from Pentacam, whereas the consistency between the two was poor (ICC = 0.442) (27). Abdi et al. reported that the WTW measured by the Sirius was significantly greater than that measured by the Pentacam, approximately 0.23 mm; there was excellent consistency between the devices (ICC = 0.95) (9). In our study, measurements from Pentacam AXL were slightly higher than those from Sirius Plus (difference of 0.06 mm), which was lower than the differences reported in previous studies and was clinically negligible. The ICC indicated excellent consistency between the two devices, suggesting that they are interchangeable for clinical practice.

The distance between the corneal endothelium and anterior surface of the lens is known as the ACD (28). ACD is an important reference for the identification of primary angle-closure glaucoma, calculation of intraocular lens power for cataract surgery, and selection of intraocular lens diameter during phakic intraocular lens implantation in eyes with a crystalline lens (29–31). Currently, the most commonly used method for ICL/TICL sizing is based on WTW and ACD. When the WTW measurement is borderline, precise measurement of ACD becomes particularly critical because an inappropriate ICL/TICL diameter may lead to secondary glaucoma or cataract formation (14). This study included the ACD of candidates for healthy refractive surgery, and significant differences were observed between the measurements obtained using the two devices. The ACD measured by the Sirius Plus device was 3.28 ± 0.21 mm, which is slightly greater than the ACD obtained from the Pentacam AXL device, which was 3.20 ± 0.22 mm. Han et al. measured the ACD of 269 healthy individuals with myopia using three devices: Pentacam, Sirius, and IOLMaster 700 (14). The average ACD measured by Pentacam, Sirius, and IOLMaster 700 were 3.26 ± 0.26 mm, 3.30 ± 0.26 mm, and 3.22 ± 0.25 mm, respectively. Furthermore, there were no significant differences between the measurements obtained by Pentacam and Sirius. This is consistent with the findings of Abdi et al., who reported an average difference of 0.01 ± 0.2 in ACD measurements between the two devices. A consistency analysis indicated that ACD values obtained from both devices are interchangeable in clinical practice (9). The discrepancies observed in our results may be attributed to differences in the sample size, ethnicity, and device models. This differs from our conclusions. Although the ICC and Bland–Altman analyses indicated that measurements from the two devices cannot be used interchangeably, the regression analysis shows good coefficients of determination. Thus, clinicians may consider calibrating for device-specific differences when using these measurements.

This study has a few limitations. First, the age distribution of the participants was concentrated among younger individuals (22.93 ± 5.53 years), which may limit the applicability of the results to older adults with cataracts. Second, cases of corneal morphological abnormalities were excluded, thereby precluding assessment of the measurement differences of the device in pathological corneas. Third, all measurements were performed by a single examiner, and the intra-observer repeatability was not assessed. Fourth, subgroup or sensitivity analysis was not conducted to identify device-specific determinants of between-device differences. Accordingly, our findings should be interpreted within the context of a healthy, young myopic population, and the interpretations regarding the mechanisms underlying the observed differences remain preliminary. Future studies should expand to multicenter designs across various age groups and include patients with pathological corneal conditions to assess the interchangeability of these devices in broader clinical scenarios. Future research should also evaluate intra-observer repeatability and inter-observer reproducibility to further validate the clinical applicability of these devices and establish a gold-standard reference system for three-dimensional parameters.

In summary, both Pentacam AXL and Sirius Plus were reliable devices for measuring anterior segment parameters in healthy populations; however, they were not interchangeable for all metrics. Despite the statistical differences, the anterior corneal surface curvature and WTW distance exhibited good consistency and correlation. Therefore, they could serve as reliable references in clinical practice.

## Data Availability

The raw data supporting the conclusions of this article will be made available by the authors, without undue reservation.
